# Interleukin‐36α suppresses growth of non‐small cell lung cancer *in vitro* by reducing angiogenesis

**DOI:** 10.1002/2211-5463.13141

**Published:** 2021-05-02

**Authors:** Xiaoxiao Xie, Haoyue Hu, Jun He, Yanyang Liu, Fengzhu Guo, Feng Luo, Ming Jiang, Li Wang

**Affiliations:** ^1^ Lung Cancer Center State Key Laboratory of Biotherapy West China Hospital of Sichuan University Chengdu China; ^2^ Department of Chemotherapy Sichuan Cancer Center School of Medicine University of Electronic Science and Technology of China Chengdu China; ^3^ Department of Oncology The Third Hospital of Mianyang (Sichuan Mental Health Center) Mianyang China; ^4^ Cancer Center State Key Laboratory of Biotherapy West China Hospital of Sichuan University Chengdu China

**Keywords:** angiogenesis, interleukin‐36α, non‐small cell lung cancer, VEGFA

## Abstract

Interleukin (IL)‐36α, a newly recognized IL‐1 family member, has been previously reported to play a pivotal role in autoimmunity diseases and acute inflammatory reactions. Recently, several studies have indicated that IL‐36α has potential anticancer effects against certain types of cancer. However, the expression pattern and functional role of IL‐36α in non‐small cell lung cancer (NSCLC) have not been elucidated. Here, we report that the mRNA and protein levels of IL‐36α are significantly reduced in NSCLC tissues. Low levels of intratumoral IL‐36α are correlated with higher tumor status, advanced TNM stage, increased vascular invasion and shorter overall survival (OS). Intratumoral IL‐36α expression is an independent prognostic factor for OS (hazard ratio = 3.081; *P* = 0.012) in patients with NSCLC. Overexpression of IL‐36α in lung cancer cells did not disturb cell proliferation, apoptosis or cell‐cycle distribution *in vitro*, but markedly inhibited tumor growth *in vivo*. Mechanistically, IL‐36α reduced the expression and secretion of vascular endothelial growth factor A through inhibiting hypoxia‐inducible factor 1α expression. Finally, decreased IL‐36α expression was associated with high microvessel density and vascular endothelial growth factor A in patients with NSCLC. Together, our findings suggest that IL‐36α expression is a valuable marker indicating poor prognosis in patients with NSCLC.

## Graphical abstract

Interleukin (IL)‐36α is an IL‐1 family member with pivotal roles in autoimmunity diseases. Here, we report that IL‐36α is a potent antitumor cytokine in non‐small cell lung cancer (NSCLC). IL‐36α suppresses tumor growth by inhibiting tumor angiogenesis via suppression of hypoxia‐inducible factor‐1α/vascular endothelial growth factor A signaling. IL‐36α expression is an independent prognostic factor for overall survival in patients with NSCLC. IL‐36α expression might be a valuable marker indicating poor prognosis in patients with NSCLC.

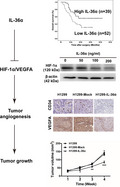

**Abbreviations**
Cat: catalog no.
CCK‐8: Cell Counting Kit‐8
EOC: epithelial ovarian cancer
HCC: hepatocellular carcinoma
HIF‐1α: hypoxia‐inducible factor 1α
HUVEC: human umbilical vein endothelial cell
IHC: immunohistochemistry
IL: interleukin
MVD: microvessel density
NF‐κB: nuclear factor‐kappa B
NSCLC: non‐small cell lung cancer
OS: overall survival
p‐: phosphorylated
rh: recombinant human
SLE: systemic lupus erythematosus
VEGFA: vascular endothelial growth factor A

Lung cancer is the leading cause of cancer‐related mortality not only in China but also around the world. Non‐small cell lung cancer (NSCLC) covers about 70–80% of all cases and includes specific pathological subtypes, such as squamous and adenocarcinoma cell carcinoma [1]. Despite significant advances in surgical resection, chemotherapy, radiotherapy, targeted therapy and novel immunotherapy, such as nivolumab (Opdivo) and pembrolizumab (Keytruda), the therapeutic effects are limited, and some patients with NSCLC experience cancer recurrence and metastasis [1, 2]. Therefore, exploring and developing new agents or therapeutic strategies for patients with NSCLC are critically important and urgent.

Interleukin (IL)‐36α (formerly named IL‐1F6) is a newly identified IL‐1 family member [3]. IL‐36α binds to the IL‐36 receptor and IL‐1 receptor accessory protein (IL‐1RAcP) to induce the downstream signaling, including adaptor protein myeloid differentiated protein 88 (MyD88), mitogen‐activated protein kinase (MAPK) and nuclear factor‐kappa B (NF‐κB) signaling pathways [4], which exert necessary roles in cell survival and differentiation. Previous studies show that IL‐36α plays prominent roles in autoimmunity diseases and acute inflammatory reactions, such as rheumatoid arthritis [5, 6], systemic lupus erythematosus [7], inflammatory bowel disease [8], primary Sjögren's syndrome [9], Graves' disease [10] and sepsis [11]. Recently, some reports have indicated that IL‐36α applies potential anticancer effects on several types of cancer, such as hepatocellular carcinoma (HCC) [12], colorectal cancer [13] and epithelial ovarian cancer (EOC) [14]. However, the functional role of IL‐36α in lung cancer and the possible underlying mechanisms remain largely unknown.

In this study, we evaluated the expression pattern and the clinical significance of IL‐36α in patients with NSCLC. Moreover, we also investigated the anticancer efficiency of IL‐36α and its possible mechanisms in lung cancer cells.

## Materials and methods

### Patients and specimens

Ninety‐one patients diagnosed with NSCLC who underwent surgery between 2014 and 2016 at Department of Thoracic Surgery, West China Hospital of Sichuan University were recruited into this study. None of them received any anticancer therapy prior to surgery. The clinicopathological characteristics, including histological types, differentiation status, smoking status and tumor TNM stages, were recorded and shown in Table 1. Tumor stages were determined by TNM classification according to the 2009 International Union Against Cancer guidelines. The histological diagnosis and grade of differentiation of the tumors were defined by evaluation of the hematoxylin and eosin‐stained tissue sections, according to the World Health Organization guidelines of classification (2009). This study was approved by the Ethics Committee of West China Hospital of Sichuan University. Written informed consent was obtained from all patients. The use of human samples complied with the standards stipulated in the Declaration of Helsinki. Tissue specimens were cut into tumor tissues and adjacent normal lung tissues (≥5 cm away from the tumor). Half of them were immediately flash frozen in liquid nitrogen for RNA and protein extraction; the remainder was fixed with formalin for immunohistochemistry (IHC).

**Table 1:** Correlation between intratumoral IL‐36α expression and clinicopathological variables in patients with NSCLC.

Variables	Cases (*N* = 91)	IL‐36α expression		*P*
		High (*n* = 39)	Low (*n* = 52)	
Age (years)				0.503
≤60	50	23	27	
>60	41	16	25	
Sex				0.180
Male	65	25	40	
Female	26	14	12	
Histological type				0.326
Adenocarcinoma	44	18	26	
Squamous cell carcinoma	32	12	20	
Others	15	9	6	
Smoking status				0.891
Smoker	67	29	38	
Nonsmoker	24	10	14	
Differentiation				0.315
High‐moderate	21	11	10	
Low	70	28	42	
Tumor status				0.012
T1–T2	60	32	28	
T3–T4	31	8	23	
TNM stage				0.006
I–II	63	33	30	
III	28	6	22	
Vascular invasion				0.019
Yes	59	20	39	
No	32	19	13	

### IHC staining and evaluation

The IHC staining was performed using microwave‐based antigen retrieval and the avidin–biotin complex method. The sections were stained with rabbit anti‐(human IL‐36α) IgG (ab180909, 1 : 1000 dilution; Abcam, Cambridge, MA, USA), rabbit anti‐(human CD34) IgG (ab81289, 1 : 1000 dilution; Abcam) or mouse anti‐(human vascular endothelial growth factor A) (VEGFA) IgG1 (ab1316, 1 : 1000 dilution; Abcam), followed by horseradish peroxidase‐labeled anti‐rabbit or anti‐mouse IgG. The sections were visualized by light microscopy using 3, 3‐diaminobenzidine.

The staining patterns of IL‐36α and VEGFA were scored based on the intensity and the percentage of positively stained cells. The intensity of the staining was scored as follows: 0 (no staining), 1 (weak staining), 2 (moderate staining) and 3 (strong staining), respectively. The staining extent was scored as follows: 0 (<10%), 1 (10–25%), 2 (25–50%) and 3 (>50%). The final score was calculated using the percentage of positive cells × staining intensity, ranging between 0 and 9. Total score ≥4 was defined as high expression, and a score of 0–3 was defined as low expression. Microvessel density (MVD) was recorded by counting CD34‐positive endothelial cells [15].

### Cell culture and transfection

Human lung cancer cell lines H1299, A549 and H1975, normal human bronchial epithelial cells, and human umbilical vein endothelial cells (HUVECs) were purchased from the American Type Culture Collection (Rockville, MD, USA) and cultured in RPMI‐1640 or DMEM (GIBCO, Shanghai, China) supplemented with 10% FBS as indicated by American Type Culture Collection. Full‐length clone cDNAs of human IL‐36α (NM_014440.1) (Sino Biological, Beijing, China) were cloned into pcDNA3.1 vector and transfected into cells using Lipofectamine 2000 (Invitrogen, Carlsbad, CA, USA). Forty‐eight hours after transfection, clone cells were selected using 600 μg·mL^−1^ G418. Successful stable transfection of overexpressing IL‐36α in lung cancer cells was assessed by real‐time quantitative PCR, western blotting and ELISA. For siRNA knockdown assay, siRNA targeting human IL‐36α (catalog no. [Cat:] sc‐72168) and scrambled siRNA were purchased from Santa Cruz Biotechnology (Santa Cruz, CA, USA). H1299 cells were transfected with IL‐36α siRNA or scrambled siRNA using Lipofectamine 3000 (Invitrogen, Life Technologies), and we evaluated knockdown efficiency after 48 h by qPCR and western blotting.

### Cell viability, apoptosis and cell‐cycle assay

Overexpressing IL‐36α‐transfected cells, mock‐transfected and normal cells were seeded into 96‐well plates and cultured for the indicated time. For cell viability assay, 10 μL Cell Counting Kit‐8 (CCK‐8) solution was added into the culture medium in each well, and absorbance (*A*) values were read using a microplate reader (Bio‐Tek Company, Winooski, VT, USA) after 2‐h incubation. For cell apoptosis assay, these cells were collected, washed twice with PBS and stained with Annexin V–FITC (5 μL) and propidium iodide (5 μL) in 500 μL binding buffer for 15 min at room temperature. For cell‐cycle assay, these cells were harvested and washed in cold PBS, fixed in 70% cold alcohol for 6 h at 4 °C and then stained with propidium iodide solution for 30 min at 4 °C. Cells in apoptosis and cell‐cycle assays were analyzed immediately by flow cytometry (BD FACSCanto; BD Biosciences, San Diego, CA, USA) and analyzed using FlowJo software (FlowJo, Ashland, OR, USA).

### Tube formation assay

HUVECs were treated with or without recombinant human (rh) IL‐36α for 8 h, then harvested and washed with PBS, and plated onto the layer of Matrigel (BD Bioscience, San Diego, CA, USA) at a density of 1 × 10^4^ cells/well. Tubular structures were quantified and photographed under a microscope after 24 h.

### RNA extraction and real‐time quantitative PCR

RNA from NSCLC tissues and cell lines was extracted using the TRIzol (Invitrogen, Carlsbad, CA, USA) method and reverse transcribed to cDNA using Moloney Murine Leukemia Virus (M‐MLV) reverse transcriptase (Invitrogen). Real‐time quantitative PCR was performed using SYBR Green PCR Master Mix (Applied Biosystems) with the following primers: *Il36α* forward: 5′‐GAACTCCACCTTCGAGTCTGT‐3′ and reverse: 5′‐CCCAAAGTCAGTAGTGTTGGC‐3′; *VEGFA* forward: 5′‐ AGGGCAGAATCATCACGAAGT‐3′ and reverse: 5′‐ AGGGTCTCGATTGGATGGCA‐3′; hypoxia‐inducible factor 1α (*Hif1*) forward: 5′‐GAACGTCGAAAAGAAAAGTCTCG‐3′ and reverse: 5′‐CCTTATCAAGATGCGAACTCACA‐3′; *Actb* forward: 5′‐AGCTTCCAGACACGCTATCAT‐3′ and reverse: 5′‐CGGTACAACGAGCTGTTTCTAC‐3′. Gene‐specific amplification was performed on ABI 7500 fast real‐time PCR system (Applied Biosystems, Foster City, CA, USA). The expression levels of VEGFA and IL‐36α were normalized to the housekeeping gene β‐actin using the comparative threshold cycle (2^−ΔΔCt^) method.

### Western blot analysis

Total protein from NSCLC tissues or cell lines was lysed using RIPA buffer with protease inhibitor (Sigma, USA). A total of 20 μg total protein was separated by 10% SDS/PAGE, transferred onto polyvinylidene fluoride membranes and incubated with rabbit anti‐(human IL‐36α) IgG (ab180909, 1 : 1000 dilution; Abcam), HIF‐1α antibody (Cat: 36169S, 1:1000 dilution; CST, Danvers, MA, USA), p38 MAPK (D13E1) (Cat: 8690S, 1 : 1000 dilution; CST), Phospho‐p38 MAPK (Thr180/Tyr182) (Cat: 9216S, 1:1000 dilution; CST), Phospho‐NF‐κB p65 (Ser536) (Cat: 3033S, 1:1000 dilution; CST), NF‐κB p65 (Cat: 8242S, 1 : 1000 dilution; CST) or β‐actin (ab119716, 1 : 1000 dilution; Abcam). The bands were then washed three times with Tris‐buffered saline with Tween 20 and probed with the horseradish peroxidase‐conjugated secondary IgG antibody for 1 h. The bands were visualized using BioImaging Systems (UVP Inc., Upland, CA, USA).

### ELISA

Cell supernatants were obtained from *in vitro*‐cultured medium by centrifuging 3000 ***g*** for 10 min at 4 ℃. VEGFA levels were quantified using a commercial human VEGFA ELISA kit (Cat: DVE00; R&D Systems, Minneapolis, MN, USA) according to the manufacturer's protocol. IL‐36α levels were examined by human IL‐36α/IL‐1F6 DuoSet ELISA (Cat: DY1078‐05, R&D Systems, Minneapolis, MN, USA) according to the manufacturer's protocol.

### *In vivo* xenograft model

For evaluation of the tumor growth *in vivo*, overexpressing IL‐36α‐transfected H1299 cells (2 × 10^6^) or A549 cells (3 × 10^6^) or related mock‐transfected cells were injected subcutaneously into the flank region of nude mice (6–8 weeks, female; Charles River Laboratories, Beijing, China). Tumor growth was monitored every week, and tumor volume was measured with digital calipers and was calculated by the following formula: tumor volume = 0.5 × width^2^ × length. After 4 weeks of tumor inoculation, tumor‐bearing mice were sacrificed. The xenografts were extracted, cut into 2‐mm^3^ cubes, fixed in 10% formalin, and embedded in paraffin. CD34‐ and VEGFA‐positive cells were detected using IHC as described earlier. The animal study was approved by the Research Ethics Committee of West China Hospital of Sichuan University. All animals received humane care according to the criteria outlined in the *Guide for the Care and Use of Laboratory Animals* prepared by the National Academy of Sciences and published by the National Institutes of Health.

### Statistical analysis

Statistical analyses were performed using the SPSS‐PC package (version 21.0; SPSS, Chicago, IL, USA). The data were expressed as mean ± standard error of the mean. The χ^2^ test was used to analyze the association between IL‐36α expression and clinicopathological variables. Overall survival (OS) was defined as the interval between surgery and death. Kaplan–Meier survival curve was plotted for the analysis of survival rates with log rank test. A multivariate Cox proportional hazards regression model was used to identify independent prognostic factors. A Student’s *t*‐test was performed to analyze differences between two groups. One‐way ANOVA was performed for three more groups. *P* < 0.05 was considered statistically significant.

## Results

### Decreased IL‐36α expression is associated with poor prognosis in patients with NSCLC

**Fig. 1:**
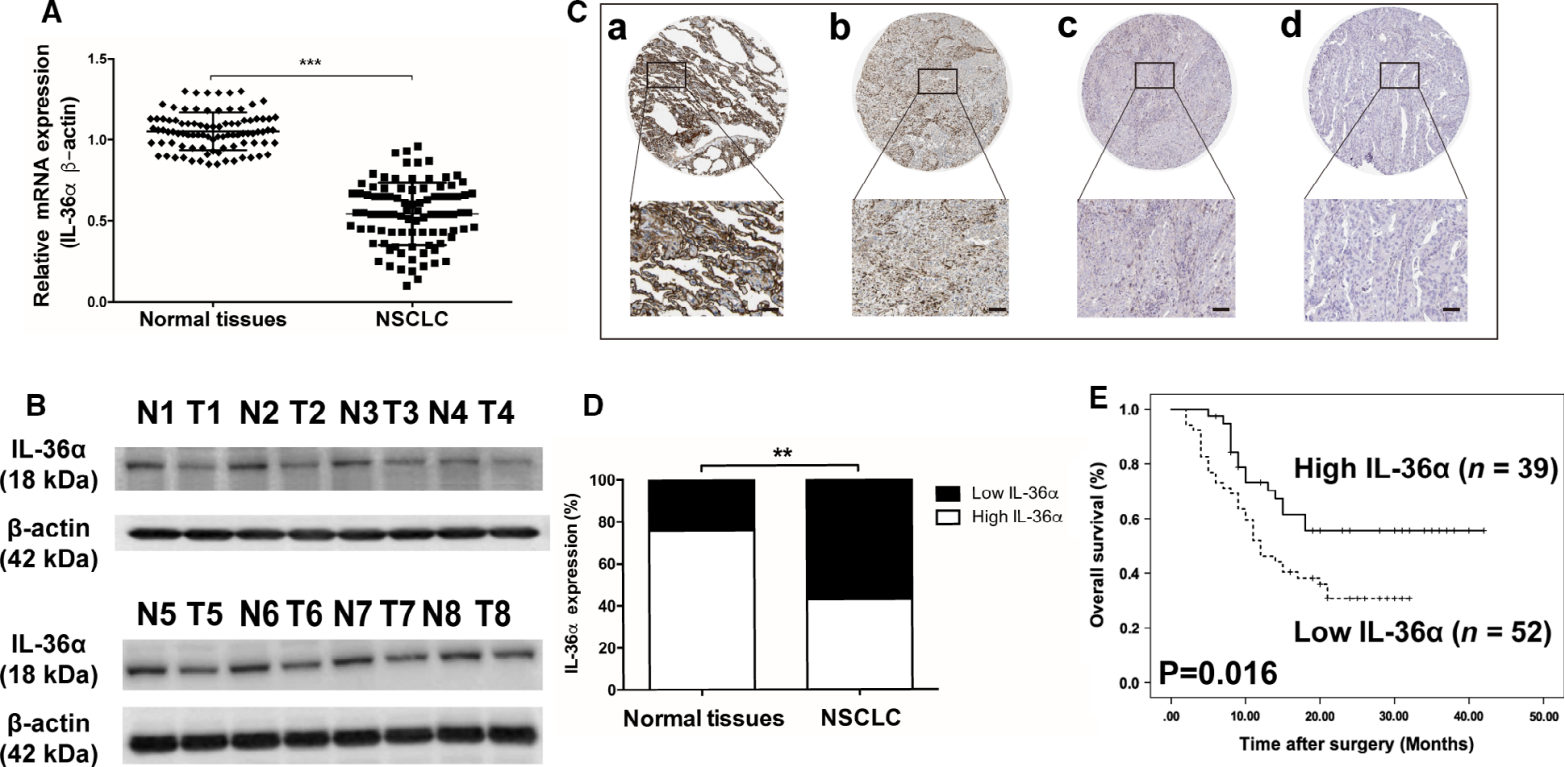
IL‐36α expression and prognosis analysis in patients with NSCLC. (A) Real‐time quantitative PCR analysis of IL‐36α expression in NSCLC tissues and corresponding normal lung tissues (*n* = 91). ***P < 0.001. (B) Western blot analysis of IL‐36α expression in NSCLC tissues and corresponding normal lung tissues (*n* = 8). (C) IHC staining of IL‐36α. High (a) staining in normal lung tissue; high (b), moderate (c) and low (c) staining in NSCLC tissues. Scale bars: 200 μm. (D) The ratio of high or low expression of IL‐36α between NSCLC tissues and normal lung tissues; **P < 0.01 (χ^2^ test). (E) Kaplan–Meier curves of survival differences among patients with NSCLC with high IL‐36α expression (*n* = 39) and low IL‐36α expression (*n* = 52) after surgery. *P* values were determined by the log rank test. Data shown are mean ± SD.

Decreased IL‐36α expression has been previously observed in HCC [12], colorectal cancer[13] and EOC [14]. However, its expression in lung cancer remains undecided. Here, we found that the mRNA and protein levels of IL‐36α in NSCLC tissues were significantly decreased compared with corresponding normal tissues (Fig. 1A,B). IHC staining further confirmed that IL‐36α was mainly located in normal lung tissues and was restricted to the cytoplasm (Fig. 1C). In addition, through IHC score, we found that IL‐36α expression in the cytoplasm was significantly lower in NSCLC tissues. As shown in Fig. 1D, the low expression of IL‐36α was covered 39 of 91 NSCLC tissues (42.8%), while high expression of IL‐36α was covered in 69 of 91 normal tissues (75.8%) (*P* < 0.01).

To investigate the prognostic value of IL‐36α in patients with NSCLC, we assessed OS for patients with high or low IL‐36α expression by Kaplan–Meier survival analysis. The results indicated that patients with NSCLC with lower cytoplasmic IL‐36α expression had significantly worse OS than those with high IL‐36α expression (*P* = 0.016, log rank test; Fig. 1E). Low IL‐36α expression was found to be significantly correlated with higher tumor status (*P* = 0.012), advanced TNM stage (*P* = 0.006) and vascular invasion (*P* = 0.019) (Table 1). Moreover, univariate and multivariate Cox regression analysis revealed that cytoplasmic IL‐36α expression was an independent prognostic factor for OS (hazard ratio = 3.081; 95% confidence interval, 1.231–3.992; *P* = 0.012; Table 2).

**Table 2:** Univariate and multivariate analyses of factors associated with OS of patients with NSCLC. CI, confidence interval.

Factors	Univariate	Multivariate		
	*P*	Hazard ratio	95% CI	*P*
Age, years (≤60 vs. >60)	0.893			
Sex (male vs. female)	0.498			
Histological type (Adenocarcinoma (ADC) vs. non‐ADC)	0.189			
Smoking status (smoker vs. nonsmoker)	0.249			
Differentiation (high‐moderate vs. low)	0.332			
Tumor status (T1–T2 vs. T3–T4)	**0.012**	1.694	0.836–2.433	**0.032**
TNM stage (I–II vs. III)	**0.006**	2.319	0.821–3.313	**0.017**
Vascular invasion (yes vs. no)	**0.021**	1.529	0.722–2.827	0.065
IL‐36α expression (high vs. low)	**0.002**	3.081	1.231–3.992	**0.012**

Bold valus are *P* < 0.05.

### IL‐36α suppresses NSCLC growth *in vivo*

**Fig. 2:**
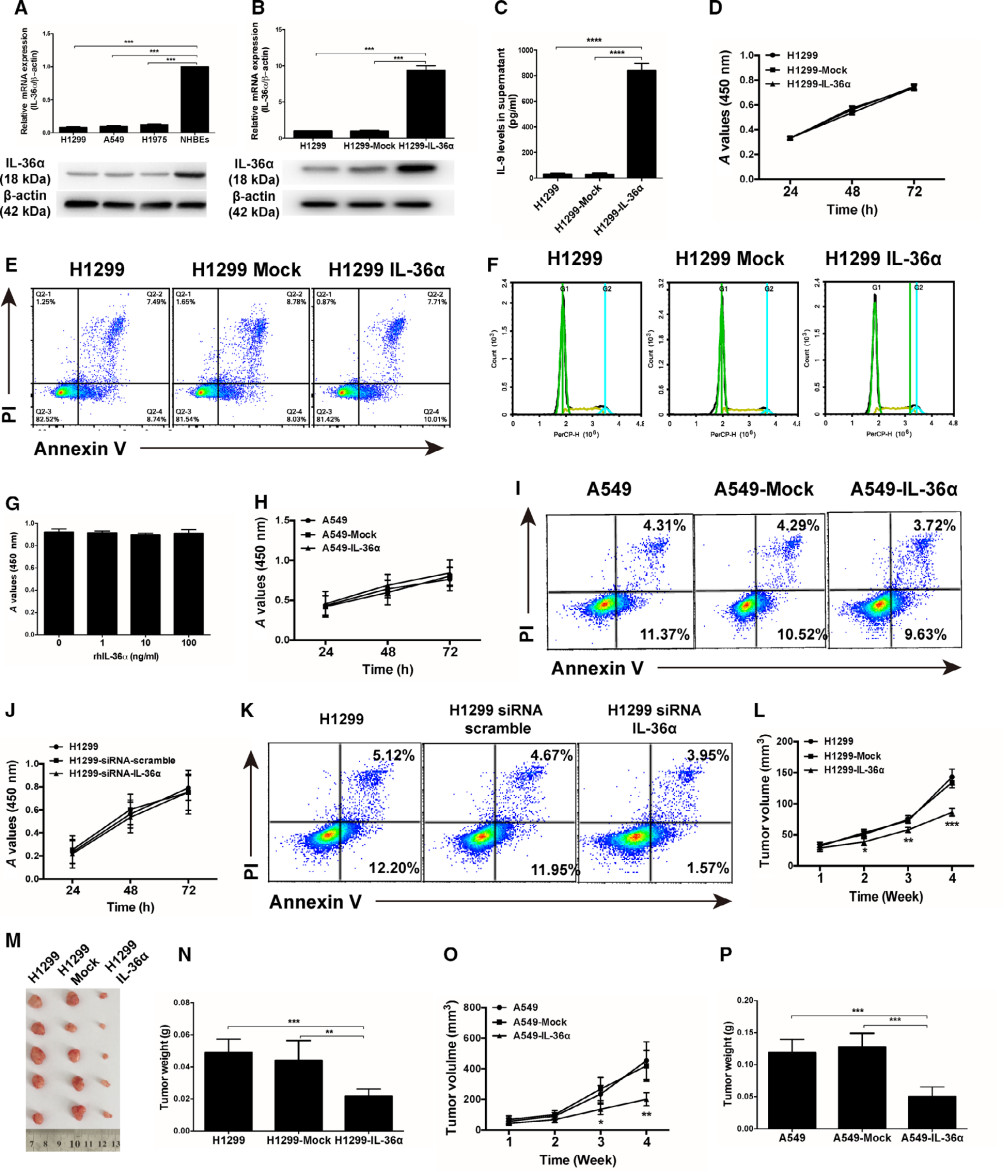
Anticancer efficiency of IL‐36α in NSCLC cells. (A) Real‐time quantitative PCR and western blot analysis of IL‐36α expression in NSCLC cell lines. (B) Real‐time quantitative PCR and western blot analysis of IL‐36α expression in control H1299, mock‐transfected H1299 and overexpressing IL‐36α‐transfected H1299 cells. (C) ELISA assay showing the protein levels of IL‐36α in control H1299, mock‐transfected H1299 and overexpressing IL‐36α‐transfected H1299 cells. (D) CCK‐8 assay analysis of cell proliferation in control H1299, mock‐transfected H1299 and overexpressing IL‐36α‐transfected H1299 cells. (E) Flow cytometry analysis of apoptosis in control H1299, mock‐transfected H1299 and overexpressing IL‐36α‐transfected H1299 cells. (F) Flow cytometry analysis of cell‐cycle arrest in control H1299, mock‐transfected H1299 and overexpressing IL‐36α‐transfected H1299 cells. (G) CCK‐8 assay analysis of cell proliferation of H1299 cells after treatment with rhIL‐36α. (H) CCK‐8 assay analysis of cell proliferation in control A549, mock‐transfected A549 and overexpressing IL‐36α‐transfected A549 cells. (I) Flow cytometry analysis of apoptosis in control A549, mock‐transfected A549 and overexpressing IL‐36α‐transfected A549 cells. (J) CCK‐8 assay analysis of cell proliferation in control H1299 cells, H1299–siRNA–scramble cells and H1299–siRNA–IL‐36α cells. (K) Flow cytometry analysis of apoptosis in control H1299 cells, H1299–siRNA–scramble cells and H1299–siRNA–IL‐36α cells. (L–N) The tumor growth curve (L), corresponding photographs of xenografts (M) and tumor weight of xenografts (N) in lung cancer‐bearing mice inoculated with H1299, mock‐transfected H1299 and overexpressing IL‐36α‐transfected H1299 cells, respectively (*n* = 5). (O, P) The tumor growth curve (O) and tumor weight of xenografts (P) in lung cancer‐bearing mice inoculated with A549, mock‐transfected A549 and overexpressing IL‐36α‐transfected A549 cells, respectively (*n* = 5). Data shown are mean ± SD from three independent experiments. *P < 0.05, **P < 0.01, ***P < 0.001, ****P < 0.0001. PI, propidium iodide.

Based on the relationship between IL‐36α and NSCLC mentioned earlier, we next explored the functional role of IL‐36α in lung cancer. First, we demonstrated that the expression levels of IL‐36α in NSCLS cell lines (H1299, A549, H1975) were decreased compared with normal lung bronchial epithelial cells (Fig. 2A). We then chose the H1299 cell line and transfected overexpressing IL‐36α in H1299 cells. The overexpression of IL‐36α was validated by qPCR, immunoblotting and ELISA (Fig. 2B,C). Through a series of analyses including CCK‐8 assay, apoptosis and cell‐cycle detection, we found that there were no significant differences in the cell proliferation, apoptosis induction and cell‐cycle arrest between overexpressing IL‐36α‐transfected H1299 cells and mock‐transfected H1299 cells (Fig. 2D–F). Notably, we also observed no effect on cell proliferation by rhIL‐36α treatment *in vitro* (Fig. 2G), suggesting that IL‐36α might not directly affect tumor growth *in vitro*. To further confirm our findings, we used another cell line, A549, to repeat the *in vitro* experiments. The data showed that overexpression of IL‐36α in A549 cells also had no effect on cell proliferation and apoptosis *in vitro* (Fig. 2H,I and Fig. S1A,B).

Our findings demonstrate that overexpression of IL‐36α did not affect cell growth of NSCLC *in vitro*. The NSCLC cells, however, expressed low levels of IL‐36α. To explore the role of endogenous IL‐36α in the growth of NSCLC cells, we knocked down the endogenous IL‐36α in H1299 cells by siRNA (Fig. S1C,D). Functional assay showed that knockdown of IL‐36α also had no obvious effect on tumor proliferation and apoptosis *in vitro* (Fig. 2J,K).

Further, we elucidated the effect of IL‐36α on NSCLC‐bearing mice *in vivo*. As shown in Fig. 2L,M, the xenografts in overexpressing IL‐36α‐transfected H1299 cell‐bearing mice grew more slowly than the mock‐transfected and normal control groups. Moreover, the tumor weights of the overexpressing IL‐36α‐transfected xenografts were markedly smaller than the mock‐transfected and normal control groups (Fig. 2N). Similar results were observed using A549 cells with overexpressed IL‐36α *in vivo* (Fig. 2O,P). Collectively, these results indicate that the anticancer effects of IL‐36α might be closely involved with the tumor growth *in vivo*.

### IL‐36α suppressed tumor angiogenesis through inhibiting HIF‐1α–VEGFA signaling

**Fig. 3:**
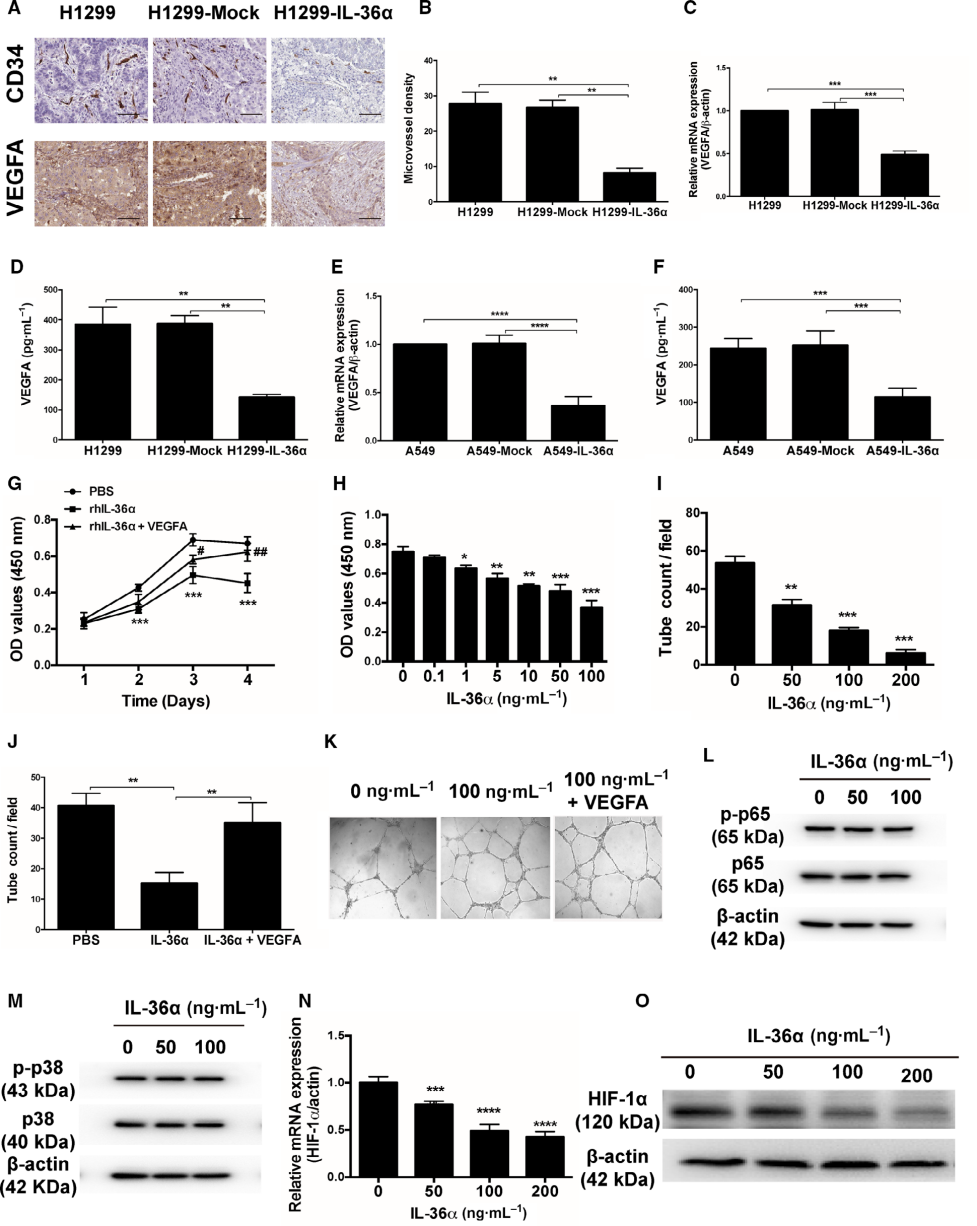
IL‐36α suppressed tumor angiogenesis through inhibiting HIF‐1α–VEGFA signaling. (A) Representative pictures of IHC staining of CD34 and VEGFA in xenografts from H1299, mock‐transfected and overexpressing IL‐36α‐transfected H1299 cells bearing mice, respectively. Scale bars: 200 μm. (B) The tumor MVD analysis through assessing IHC staining of CD34 in xenografts from H1299, mock‐transfected and overexpressing IL‐36α‐transfected H1299 cells bearing mice, respectively. (C, D) VEGFA levels detection in H1299, mock‐transfected and overexpressing IL‐36α‐transfected H1299 cells through real‐time quantitative RT‐PCR and ELISA. (E, F) VEGFA levels detection in normal A549 cells, mock‐transfected A549 cells and overexpressing IL‐36α‐transfected A549 cells through real‐time quantitative RT‐PCR and ELISA. (G, H) CCK‐8 assay analysis of HUVEC proliferation in the presence of rhIL‐36α with or without VEGFA (100 ng·mL^−1^). (I–K) The effect of rhIL‐36α on capillary structure formation of HUVECs with or without VEGFA (100 ng·mL^−1^). Original magnification ×200. (L, M) The protein levels of p‐P65, p65, p‐p38 and p38 in HUVECs treated with rhIL‐36α. (N) The mRNA levels of HIF‐1α in HUVECs treated with rhIL‐36α. (O) The protein levels of HIF‐1α in HUVECs treated with rhIL‐36α. Data shown are mean ± SD. *P < 0.05, **P < 0.01, ***P < 0.001, ****P < 0.0001, compared with PBS; ^#^ *P* < 0.05, ^##^ *P* < 0.01, compared with rhIL‐36α + VEGFA.

Angiogenesis contributes to tumor growth, and VEGFA is a potent inducer of angiogenesis *in vivo* [16]. We speculate that IL‐36α‐suppressed tumor growth of NSCLC *in vivo* might be associated with reducing angiogenesis. As expected, we found that overexpressing IL‐36α‐transfected xenografts had lower CD34 expression, which indicates the MVD and VEGFA expression compared with mock‐transfected and normal control groups (Fig. 3A,B). Moreover, we found that VEGFA mRNA and protein levels were also significantly reduced in overexpressing IL‐36α‐transfected H1299 xenografts and A549 xenografts, respectively (Fig. 3C–F). Furthermore, we found that rhIL‐36α treatment could significantly inhibit HUVEC growth in a time‐ and dose‐dependent manner (Fig. 3G,H) and repress the formation of vessel‐like tubes in a dose‐dependent manner (Fig. 3I). Importantly, these effects could be abolished by adding VEGFA protein (Fig. 3J,K), suggesting that IL‐36α suppresses tumor angiogenesis by regulating VEGFA expression.

IL‐36α binds to the IL‐36 receptor and initiates signal transduction and activation through NF‐κB and MAPK pathways [17]. However, we found that the expressions of phosphorylated (p)‐p65 and p‐p38 MAPK were not changed after IL‐36α treatment in HUVECs (Fig. 3L,M), suggesting that IL‐36α inhibits angiogenesis in a NF‐κB and/or MAPK signaling‐independent manner. HIF‐1α is a critical transcription factor that regulates VEGFA expression [18]. We found that the mRNA and protein levels of HIF‐1α were significantly reduced after IL‐36α treatment in HUVECs (Fig. 3N,O), indicating that IL‐36α suppresses VEGFA production by down‐regulation of HIF‐1α expression.

### Decreased IL‐36α expression is associated with high MVD and VEGFA in patients with NSCLC

**Fig. 4:**
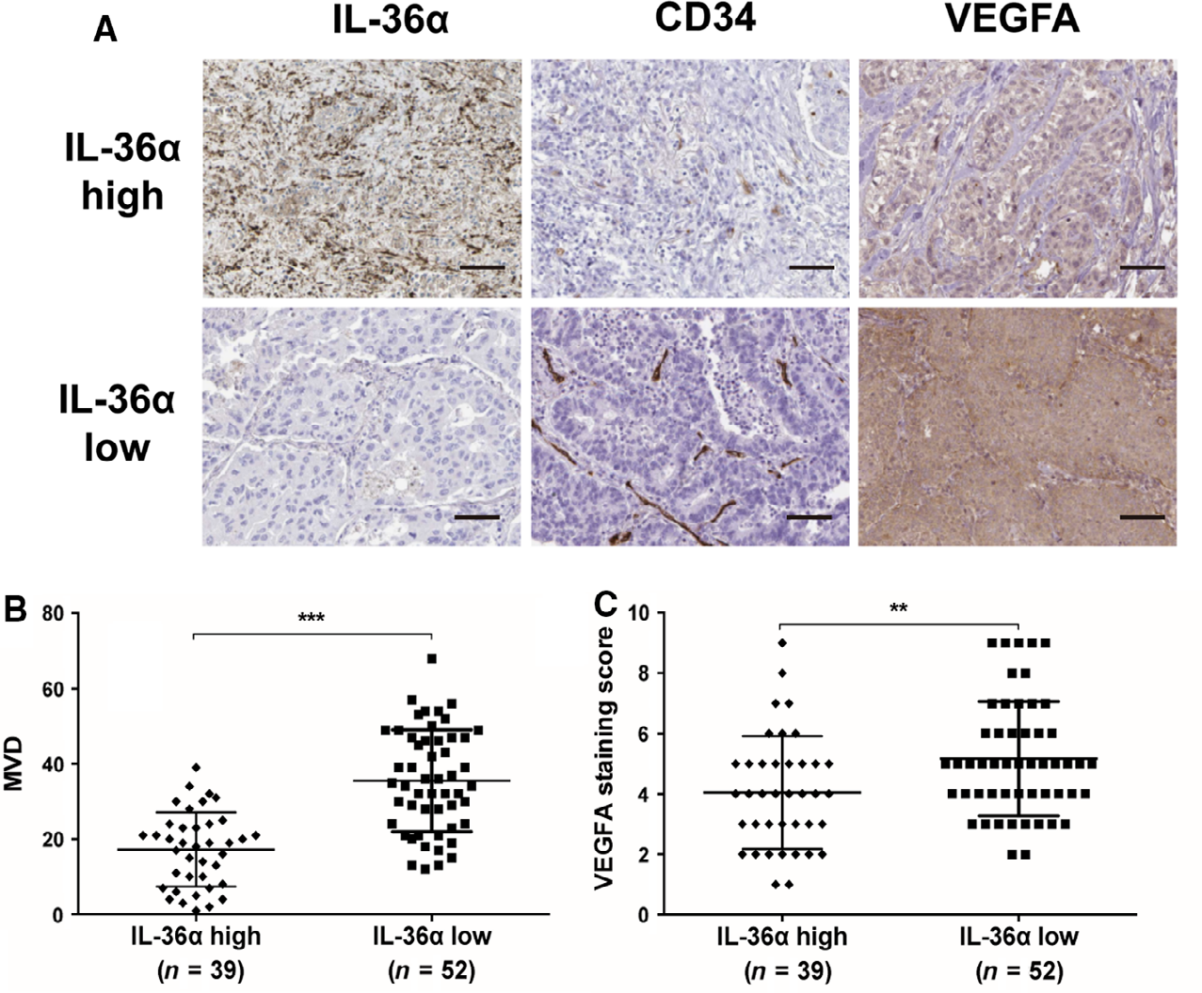
IHC staining of IL‐36α, CD34 and VEGFA in patients with NSCLC. (A) Representative IHC staining of IL‐36α, CD34 and VEGFA in NSCLC tissues of patients with high or low expression of IL‐36α. Scale bars: 200 μm. (B, C) The correlation analysis of MVD and VEGFA expression in patients with NSCLC with high or low expression of IL‐36α. Data shown are mean ± SD. **P < 0.01, ***P < 0.001.

To further confirm the functional role between IL‐36α and angiogenesis in patients with NSCLC, we analyzed 91 NSCLC tissues through IHC staining with CD34 and VEGFA. The results demonstrated that those patients with low IL‐36α expression exhibited higher MVD levels (Fig. 4A,B), as well as higher VEGFA expression (Fig. 4A,C), compared with patients with high IL‐36α expression, suggesting that IL‐36α expression might be negatively associated with MVD and VEGFA levels in patients with NSCLS.

## Discussion

Recent reports demonstrate that IL‐36α exerts potential anticancer function in certain types of cancer, including HCC, colorectal cancer and EOC. However, there is little knowledge about the relationship between IL‐36α and NSCLC. Here, we provided the evidence that IL‐36α might be a poor prognosis marker for patients with NSCLC and play potent anticancer efficiency through suppressing tumor angiogenesis.

In this study, we first investigated the expression pattern of IL‐36α in NSCLC tissues of patients and analyzed its clinical significance based on the supported data, including histological types, tumor status and TNM stage. Our results demonstrated that IL‐36α, located in cytoplasm, was mainly expressed in nontumor tissues and was decreased in NSCLC tissues. In addition, reduced IL‐36α expression was observed to be remarkably negatively associated with higher tumor status, advanced TNM stage and vascular invasion. Patients with low cytoplasmic IL‐36α expression were associated with poor overall prognosis. Our findings are consistent with prior studies, where IL‐36α expression was decreased and closely associated with tumor progression in HCC [12], colorectal cancer [13] and EOC [14].

Next, the functional role of IL‐36α on lung cancer cells was explored. As expected, the expression levels of IL‐36α were decreased in NSCLC cells compared with the normal lung bronchial epithelial cells, which was similar with our previous results verified in NSCLC tissues. Through transfecting overexpressing IL‐36α in lung cancer cells, we did not observe significant effects of IL‐36α on the cell proliferation, apoptosis induction and cell‐cycle arrest. Indeed, in HCC, Pan et al. [12] also clarified that there was no significant difference of cell proliferation *in vitro* between overexpressing IL‐36α‐transfected HepG2 cells and the control vector. On the contrary, Chang et al. [14] reported that overexpressing IL‐36α suppressed proliferation of EOC cells *in vitro*, which indicated that the functional role of IL‐36α might be of difference in diverse types of tumors. Interestingly, in lung cancer‐bearing mice, the growth of overexpressing IL‐36α‐transfected xenografts was suppressed obviously compared with the mock‐transfected and control groups. Based on our previous results mentioned earlier, we speculate that IL‐36α‐inhibited tumor growth is possibly associated with the regulation of the tumor microenvironment.

VEGFA is one of the most potent proangiogenic factors, which is closely involved with angiogenesis in the tumor microenvironment and significantly promotes tumor growth [19]. In our next study, through IHC staining, we found that IL‐36α was negatively correlated with MVD and VEGFA expression in tumor tissues of lung cancer‐bearing mice and NSCLC tissues. In addition, in overexpressing IL‐36α‐transfected H1299 cells, the expression and secretion of VEGFA had decreased. This effect was further verified by the time‐ or dose‐ dependent proliferation and formation of vessel‐like tubes of HUVECs *in vitro* treated with rhIL‐36α, indicating that IL‐36α regulated tumor angiogenesis by targeting VEGFA.

## Conclusion

In summary, our findings demonstrate that IL‐36α expression is associated with poor prognosis in patients with NSCLC, and IL‐36α exerts potent anticancer efficiency in NSCLC involved with, or partly, reducing tumor angiogenesis via inhibiting VEGFA expression. Therefore, IL‐36α might be a valuable prognostic marker and therapeutic target for patients with NSCLC.

## Conflict of interest

The authors declare no conflict of interest.

## Author contributions

XX conceived and designed the study. HH, JH, YL and FG carried out the study and analyzed the data. XX, MJ, FL and LW wrote the manuscript. All authors read and approved the final manuscript.

## Supporting information


**Fig. S1.** The expression of IL‐36α in knockdown or overexpression of lung cancer cell lines.

## Data Availability

The data that support the findings of this study are available upon request from the corresponding author.
