# Supplementary figures and images for: Interleukin‐36α suppresses growth of non‐small cell lung cancer in vitro by reducing angiogenesis
Source: FEBS Open Bio. 2021 May 2;11(5):1353–63. doi: 10.1002/2211-5463.13141 (PMC8091581; doi:10.1002/2211-5463.13141)

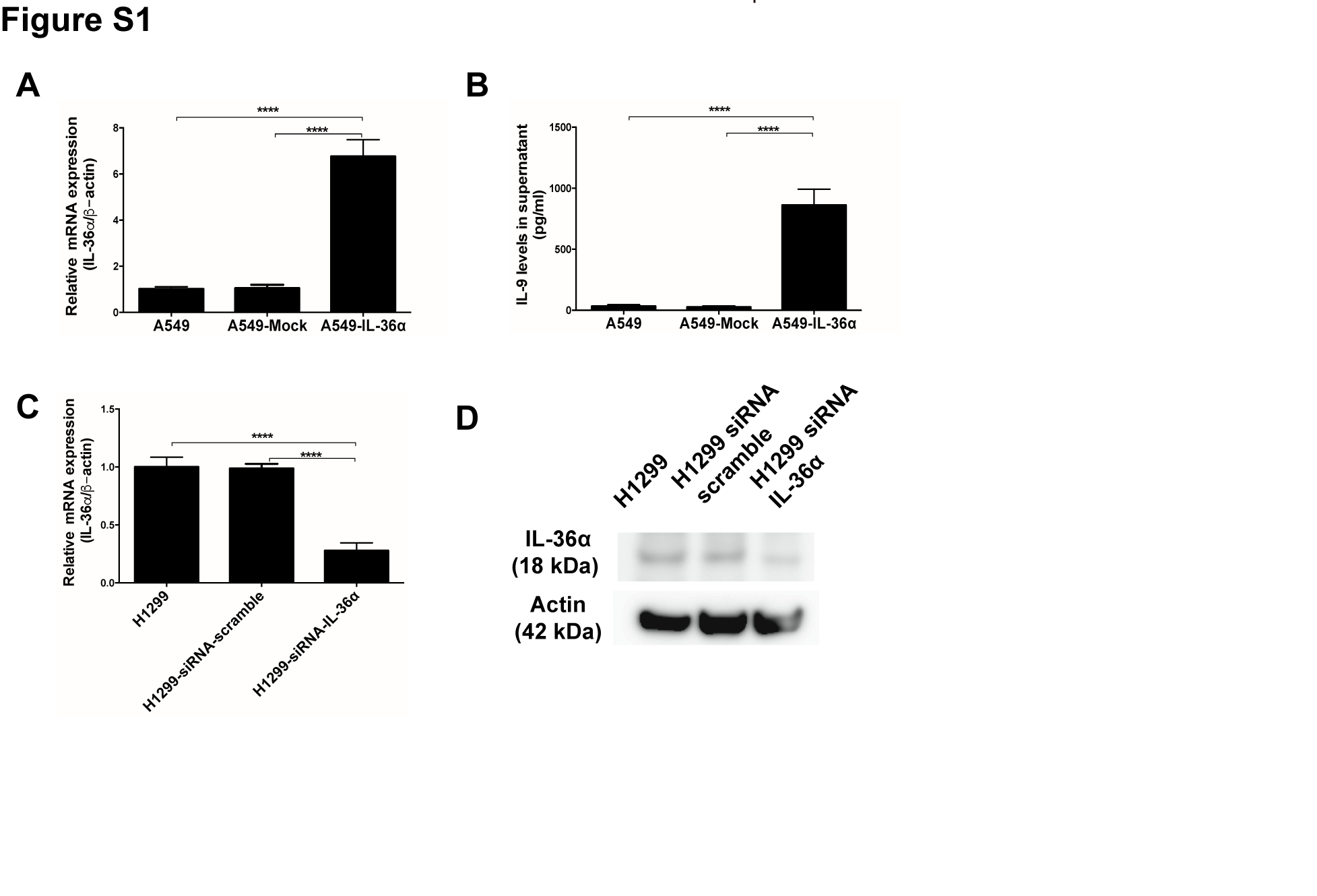

Supplement: Supplementary file 1 — Fig. S1. The expression of IL‐36α in knockdown or overexpression of lung cancer cell lines. [file FEB4-11-1353-s001.tif]
